# Current Trends on Mechanical, Corrosion Resistance, and Antibacterial Properties of Metallic Materials

**DOI:** 10.3390/ma15113822

**Published:** 2022-05-27

**Authors:** Marjetka Conradi, Aleksandra Kocijan

**Affiliations:** Institute of Metals and Technology, Lepi pot 11, 1000 Ljubljana, Slovenia; aleksandra.kocijan@imt.si

The scope of the Special Issue entitled “Mechanical, Corrosion Resistance, and Antibacterial Properties of Metallic Materials” includes research regarding the latest developments in materials’ mechanical properties and characterization, pure/applied corrosion phenomena, and advanced understanding of bacterial adhesion and the induced antibacterial properties of metallic materials.

In the field of metallic materials, improvements in the technological properties of materials, their functionality, and the life span of products are increasingly required. Metallic materials are used in most engineering applications that require high corrosion resistance combined with favorable mechanical properties [1,2]. The protection of metallic surfaces from corrosion represents one of the most important steps in satisfying the needs of demanding industrial applications. The general aim is to prolong the life span of metallic parts used in various industrial components and to reduce the total costs and environmental problems connected to material failure [3]. The durability of engineering materials in terms of friction/wear and corrosion is governed by the materials’ surface rather than bulk properties [4]. Surface modification is therefore necessary to improve the materials´ performance while maintaining the required bulk characteristics, which can further expand their application possibilities. In the past few decades, various techniques, such as the application of polymer coatings [5], electron-beam lithography [6], plasma treatment [7,8], self-assembly techniques [9] and laser surface processing [10], were introduced to improve the surface properties of metallic materials. A variety of polymers have been known to be useful in metallic surface protection, including epoxy resins, polyesters and polyurethanes [11,12,13]. To further improve the corrosion properties of polymer coatings and their mechanical properties, recently, different inorganic nanoparticles have been successfully incorporated into polymer matrices [14,15]. This offers an environmentally friendly method to enhance the integrity and durability of coatings, as finely dispersed particles in coatings can fill cavities and cause crack deflection or crack bridging. Therefore, they provide an effective physical barrier between the substrate and the environment and serve as a reservoir for corrosion inhibitors that help metallic surfaces to more effectively resist attacks from aggressive species.

Furthermore, in biomedical applications, when a material is exposed to physiological conditions, multiple aspects of its surface properties, including wettability, surface energy and topography, need to be evaluated. The biocompatibility of a material is related to cell behavior upon contact with the surface. The surface characteristics of materials, such as surface topography, chemistry or surface energy, have essential roles in the adhesion process. The quality of this first phase of cell–material interaction influences and enables the good proliferation and differentiation of the cells on the surface [16]. Material biocompatibility is significantly influenced by the surface topography, as cell attachment and proliferation can be controlled through micro/nanoscale roughness by mimicking the natural environment at the molecular level [17]. Improved cell attachment and resistance to bacterial adhesion are two critical characteristics that should be considered in an effective biomaterial design. The appropriate surface modification of the substrate, as mentioned in the previous paragraph, is also crucial for a desirable biological response. Different topographic patterns influence the size, shape and spatial distribution of the attached cells [18]. In addition, wettability also has an important role in cell adhesion [19]. It has been shown that surfaces with moderate wettability have a higher rate of cell attachment than superhydrophilic and superhydrophobic surfaces [20,21]. It has also been shown that interactions between topographic patterns and bacterial adhesion cannot be generalized, as the size and the shape of bacteria are crucial for their spatial distribution. An increased surface roughness has been shown to be associated with improved cell integration. Additionally, most studies have indicated a positive correlation between a large surface area with a rough topography and the amount of adhering bacteria [21,22].

In conclusion, this Special Issue represents an important contribution to the broader field, which will result in an increased number of article reads and citations. It presents research that uses novel approaches to investigate the basic material properties and microstructures that affect the mechanical properties, corrosion resistance and antibacterial properties of metallic materials. The manuscripts included reflect recent developments in the science and technology of metallic materials through the latest scientific research achievements in new processes and their dissemination and applications. The Special Issue focuses on the advanced understanding of the structural and mechanical properties of metallic materials as well as the discovery, development and/or characterization of the structure of improved metallic materials with novel functional or mechanical properties of potential engineering interest. This Special Issue presents the latest developments in the fields of corrosion mechanisms and corrosion control, passivity, anodic oxidation and biochemical corrosion, as well as improvements in the antibacterial properties of metallic materials caused by changing the chemical composition of metallic materials or by the application of specific coatings.
